# Diversity of life history and population connectivity of threadfin fish *Eleutheronema tetradactylum* along the coastal waters of Southern China

**DOI:** 10.1038/s41598-023-31174-x

**Published:** 2023-03-09

**Authors:** Zhongya Xuan, Wen-Xiong Wang

**Affiliations:** 1 School of Energy and Environment and State Key Laboratory of Marine Pollution, City University of Hong Kong, Kowloon, Hong Kong China; 2 Research Centre for the Oceans and Human Health, City University of Hong Kong Shenzhen Research Institute, Shenzhen, 518057 China

**Keywords:** Biogeography, Conservation biology, Population dynamics

## Abstract

Understanding the diversity of life history, life stage connectivity and population is essential to determine the spatial scale over which fish populations operate. Otolith microchemistry analysis is a powerful tool to elucidate the life history and population connectivity of fish, providing important insights to the natal origin and population structure. In this study, we used laser ablation inductively coupled plasma mass spectrometry to analyze the chemical composition of otoliths throughout the entire lifetime of endangered fourfinger threadfin species, *Eleutheronema tetradactylum*. We reconstructed the life history of *E. tetradactylum* from Southern China collected from different locations over a spatial scale of 1200 km. Sr:Ca and Ba:Ca ratios profiles from otolith core-to-edge analysis suggested two contrasting life history patterns. Based on the differences in early life stages, we identified some fish spending their first year in an estuarine environment with subsequent movement to marine coastal systems, while some fish remaining in the coastal systems throughout their entire early life history stages. The non-metric multi-dimensional scaling showed a strong overlap in otolith core elemental composition, indicating a large-scale connectivity in the life history of *E. tetradactylum*. The immature fish from different natal origins mixed to a large extent when they fed and overwintered in the extensive offshore waters. Clustering of near core chemistry pointed to three possible sources of nursery for the threadfin fish. This study demonstrated the diversity of life history patterns of *E. tetradactylum* in Southern Chinese waters. Restoration in egg and larvae densities in coastal waters and estuaries may enhance their population abundances.

Many fishes have complex life history and often exhibit variations in habitat usages at different life history stages. They undertake ontogenetic movements to allow access to a variety of habitats and resources^1,2^. Identification of life stage connectivity between essential habitats of species is thus a key to facilitate our understanding of their ecological behavior and design appropriate management and conservation measures^3–5^. The life history diversity is constituted by the difference of whether and how long the different habitats are used, which have profound effects on species distribution, abundance, and resilience to changing environmental conditions^6,7^. The asynchronous dynamics among different life histories may stabilize the populations through portfolio effects^8^. Therefore, a high level of intrapopulation life history diversity can increase the persistence and resilience of population facing variable environmental conditions and anthropogenic impacts.

Population connectivity defines the exchange of larvae, juveniles or adults among geographically separated groups across a species range^9,10^. For marine animals with a planktonic stage, larval transport provides a unique opportunity for dispersal, allowing adult fish to cross broad geographic areas (e.g., species distribution) as well as mix between geographically segregated populations^11,12^. The degree of connectivity influences the fundamental processes such as population dynamics and community composition^10,11^. As such, understanding the extent of population connectivity in fish species is essential to determine the spatial scale over which fish populations operate their structures, and is paramount to apply adapted management measures for metapopulation persistence.

Due to the small size and high mortality of larvae and juveniles, artificial tags such as mark-recapture, passive integrated transponder tags, or radio-telemetry are unable to provide lifetime insights for identifying fish movement in different aquatic environments. Otoliths chronologically precipitate calcium carbonate onto the organic matrix across their entire lifespans, and the increments are not metabolically reworked or resorbed after deposition^13,14^. It has been demonstrated that certain elements (mainly Sr and Ba) substituted for calcium directly in crystal structure, and their concentrations in otolith were associated with the concentrations of these elements in the ambient water^14–16^. Although some processes (e.g., reproduction, growth) may affect the otolith chemistry^17,18^, many studies showed that Sr:Ca and Ba:Ca concentrations in otoliths were correlated with those in the surrounding environment^19,20^. Consequently, by analyzing the elemental concentrations of otolith across its entire growth axis (core to edge), it is possible to reconstruct the past environmental conditions in which fish lived during their entire life cycle.

The fourfinger threadfin, *Eleutheronema tetradactylum* (Shaw 1804), belongs to the Polynemidae family, and is widespread along the Indo-West Pacific coast, ranging from the Papua New Guinea and northern Australia to Persian Gulf^21^. This species is a commercially important species in many countries due to its remarkable meat quality^22^, and have major economic and culture values along the southern coast of China, with a high price in local fish markets (e.g., one kilogram of wild caught fish costed close to 100 US dollars in Hong Kong). *E. tetradactylum* is a protandrous hermaphrodite with male individuals undergoing sex change to female at different ages^23^. Its four threadlike independent pectoral fin rays move independently from the unmodified fin, and display tactile functions by acting as sensory probes for locating food in muddy habitats^24^. However, due to the commercial over-exploitation and many other anthropogenic impacts, populations of *E. tetradactylum* declined rapidly across much of its Indo-West Pacific range over the past decades, and this fish is now classified as endangered by the International Union for Conservation of Nature (IUCN).

Understanding the life history and population structure of fish is essential to generate management actions for recovering the resource in the future. Previous studies suggested that *E. tetradactylum* generally lived in continental shelves on shallow muddy and sandy substrata, and the larvae and juveniles were found in brackish waters of estuarine systems (with lack of information about spawning)^25^. Some studies found that adult *E. tetradactylum* used coastal intertidal habitats^26,27^. Although some studies investigated the large-scale habitat utilization, the fine-scale behavior of individuals, diversity of habitat use, contribution of source areas of juveniles to adult population, and the degree of connectivity among geographically separated groups, remain essentially unexplored. Earlier, we utilized otolith chemistry to describe the life histories of *E. tetradactylum* from each of one location in Thailand and China^28^, and found that the majority of *E. tetradactylum* individuals were coastal-dependent, i.e., remaining in the coastal systems throughout their early life history stages as their nursery. However, in the middle and lower Merbok River estuary (north-west of Peninsular Malaysia), *E. tetradactylum* larvae were identified by the DNA metabarcoding^25^. In the present study, we attempted to use otolith elemental signatures as natural tags to detect the diversity of their life history in different geographical groups, and to evaluate the connectivity between nursery areas and catch locations. Our objectives were to 1) quantify the otolith Sr:Ca and Ba:Ca concentrations across life-time elemental profiles, to infer whether there was transition between estuarine and coastal waters and evaluate the diversity of migratory strategies; 2) examine the timing of potential transitions between estuarine and coastal waters during early life history; 3) examine the contribution of potential nursery areas to the catch locations, and the degree of connectivity among catch groups.

## Materials and methods

### Study area and fish sampling

**Figure 1:**
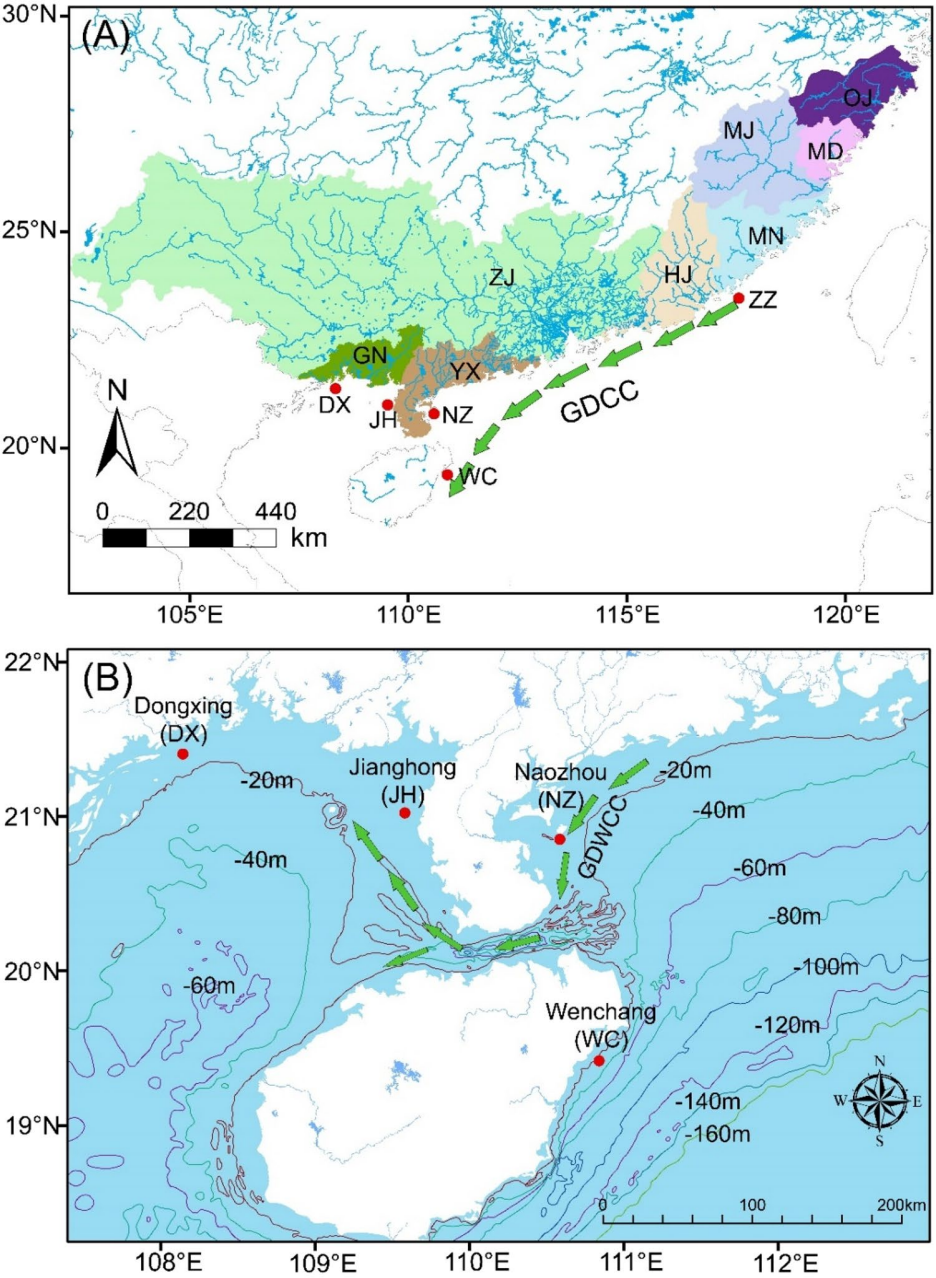
The locations where the fourfinger threadfin, *Eleutheronema tetradactylum*, were collected. (**A**) The different colored areas on the land represent the different basins along the southern coast of China, Oujiang Basin (OJ), Mindong Basin (MD), Minjiang Basin (MJ), Minnan Basin (MN), Hanjiang Basin (HJ), Zhujiang Basin (ZJ), Yuexi Basin (YX), Guinan Basin (GN). Red dots show the sample sites. (**B**) The green arrow symbol is the schematic representation of currents. The map was drawn using open source software QGIS 2.14 (http://www.qgis.org/). The base map was downloaded from the Natural Earth (open access) at https://www.naturalearthdata.com/. The boundary of different basins was extracted from HydroBASINS at https://www.hydrosheds.org/products/hydrobasins.

A total of 66 *Eleutheronema tetradactylum* individuals were collected through commercial fishing in Southern China from five locations: Zhangzhou (ZZ), Naozhou (NZ), Jianghong (JH), Dongxing (DX), and Wenchang (WC) (Table 1, Fig. 1), across a spatial scale of 1200 km. The samples from NZ were collected twice (Nov. 2020 and Nov. 2021), whereas the sampling from other four locations were collected in Nov. 2021. Fourfinger threadfin individuals were sampled by commercial fishery boats. Due to the high price in local fish markets and vulnerable state of this endangered species, the number of samples in this study was relatively low. Nonetheless, some previous studies demonstrated the efficacy of otolith microchemistry in revealing fish life history and population structure based on small sample sizes^29,30^. Although previous studies reported *E. tetradactylum* in the East China Sea and the South China Sea^31,32^, our survey on the important fishing ports along the coasts of China from Yangtze River estuary to Guangxi only found *E. tetradactylum* at the coasts of Southern China. Based on the von Bertalanffy growth curve of *E. tetradactylum* fitted by the previous study^33^, we used the total length of each specimen in this study to estimate the age of *E. tetradactylum* samples, and all individuals were of the same age (4 +).

**Table 1:** Sampling locations and sample characteristics of fourfinger threadfin, *Eleutheronema tetradactylum*, collected from China.

Location	Sampling date	Population code	Number	Total length (cm, mean ± SD)
Zhangzhou	2021.11	ZZ	7	62.2 ± 2.36
Naozhou 2021	2021.11	NZ-21	13	60.3 ± 4.12
Naozhou 2020	2020.11	NZ-20	15	61.3 ± 3.97
Jianghong	2021.11	JH	15	59.0 ± 3.57
Dongxing	2021.11	DX	7	61.0 ± 2.00
Wenchang	2021.11	WC	9	62.8 ± 4.32

DX was located on the westernmost coast of China, and JH and NZ were located on the west and east sides of Leizhou Peninsula in Southern China, respectively. DX and JH were both adjacent to the Beibu Gulf, and NZ was located on the western Guangdong where water exchange between the coastal waters of western Guangdong and Beibu Gulf took place through the Qiongzhou Strait. The Guangdong Western Coastal Current (GDWCC) moves westward during most of the year but shifts eastward during the monsoon season^34,35^. WC was located on the east side of Hainan island. ZZ was adjacent to the Taiwan Strait, and distant from other sites (~ 1200 km distance from DX, ~ 900 km distance from WC). During winter the Guangdong Coastal Current (GDCC) from Guangdong moves westward to the southeast of Hainan island^34^.

**Figure 2:**
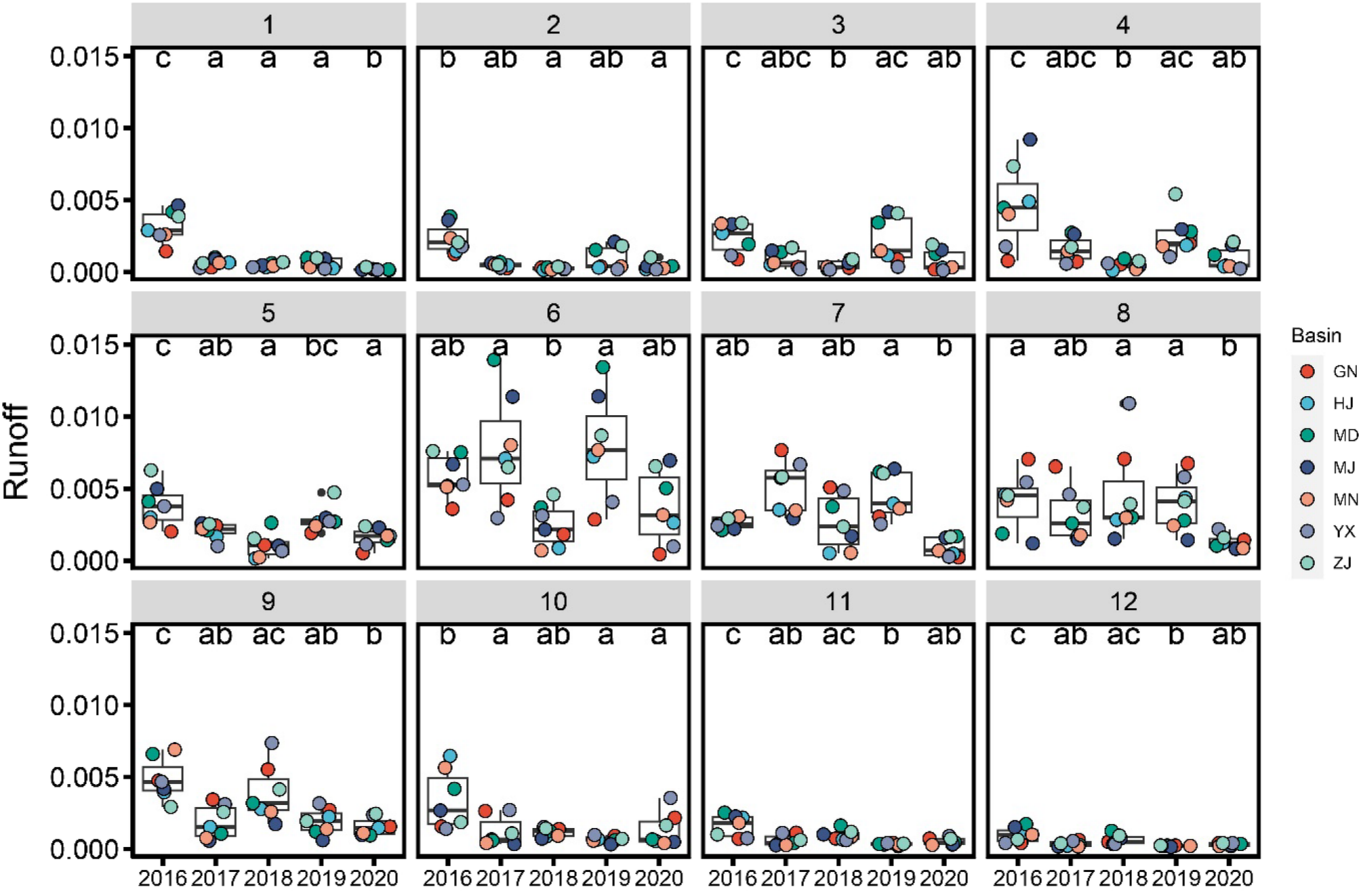
Average monthly runoff of the basins throughout coast of Southern China from January of 2016 to December of 2020. The 12 facets represent the months from January to December. Horizontal line in box: median value; bottom and top of box: 25th and 75th percentiles; whiskers: 5th and 95th percentiles.

As estuaries are the transition zones linking freshwater and marine environments, their environmental and hydrological processes are largely governed by the degree of freshwater inflow^36,37^. Therefore, the freshwater inflow may be an important environmental variable affecting fish recruitment^36,38,39^. Freshwater inflow to an estuary is usually related to runoff directly, although there are estuaries that receive freshwater from springs^36^. In this study, runoff in coastal basins throughout the coast of Southern China was used as a proxy for freshwater inflow to assess the interannual variation in habitat availability (the boundary of the basin is shown in Fig. 1). We accessed ERA5-Land ECMWF Climate Reanalysis to extract the regional average monthly runoff data from January of 2016 to December of 2020^40^. To make the data comparable across years and basins, we used the average values of runoff of each basin (Fig. 2).

### PCR sequencing and evaluation of population genetic structure

The captured fish were immediately preserved on ice and stored frozen at -20 °C until processing in the laboratory. For the molecular analysis, about 200 mg muscle tissues of each fish were collected and preserved in 95% ethanol, and stored at -20 °C until DNA extraction. For all specimens, genomic DNA was extracted from the muscle using a commercial DNA extraction kit (TIANGEN Biotech) following the manufacturer’s protocol. Total length (TL, to nearest mm) of fish was recorded. The specimen was first identified up to the species level with morphological methods, followed by the mtDNA gene, cytochrome C oxidase I (COI), as a genetic marker for species identification. To amplify the partial mtDNA COI fragment, PCR was carried out using the previously universal primers for COI, following the conditions as described^41^. Each 25 μL PCR reaction system contained 2.5 μL of 10 × Buffer, 2.0 μL of dNTPs, 0.5 μL forward primer (10 mM), 0.5 μL backward primer (10 mM),0.5 μL of template DNA, 0.2 μL of Taq (TaKaRa), and 18.8 μL of ddH_2_O. The PCR thermocycling conditions were as follows: initial denaturation at 94 °C for 4 min followed by 30 cycles of denaturation at 94 °C for 30 s, annealing at 50 °C for 30 s, extension at 72 °C for 45 s, and final extension at 72 °C for 5 min. The quality of each PCR product was assessed by 1% agarose gel electrophoresis and observed under the UV light. All qualified PCR products were submitted for sequencing. Each COI sequence were aligned using Clustal W, and the alignments were subjected to population genetic structure analysis.

The level of genetic divergence between populations was evaluated by the genetic differentiation index (FST) between each pair of populations using Arlequin 3.5. The statistical significances of the pairwise FST values were evaluated through 1000 permutations. The haplotype network was constructed based on median-joining algorithm in PopART 12 to estimate the gene genealogies at the population level.

### Otolith microchemistry

Otolith preparation was conducted following the established protocols^28^. In the laboratory, sagittal otoliths were extracted from each fish and rinsed with deionized water to remove any adhering tissues. After dried in the air, the left sagittal otoliths were embedded in Epofix epoxy resin (Struers, Copenhagen, Denmark), and later sectioned across the core. Each otolith section was mounted on glass slides and ground by hand with a series of waterproof abrasive grinding papers to expose the core, and further polished with silica suspension.

**Figure 3:**
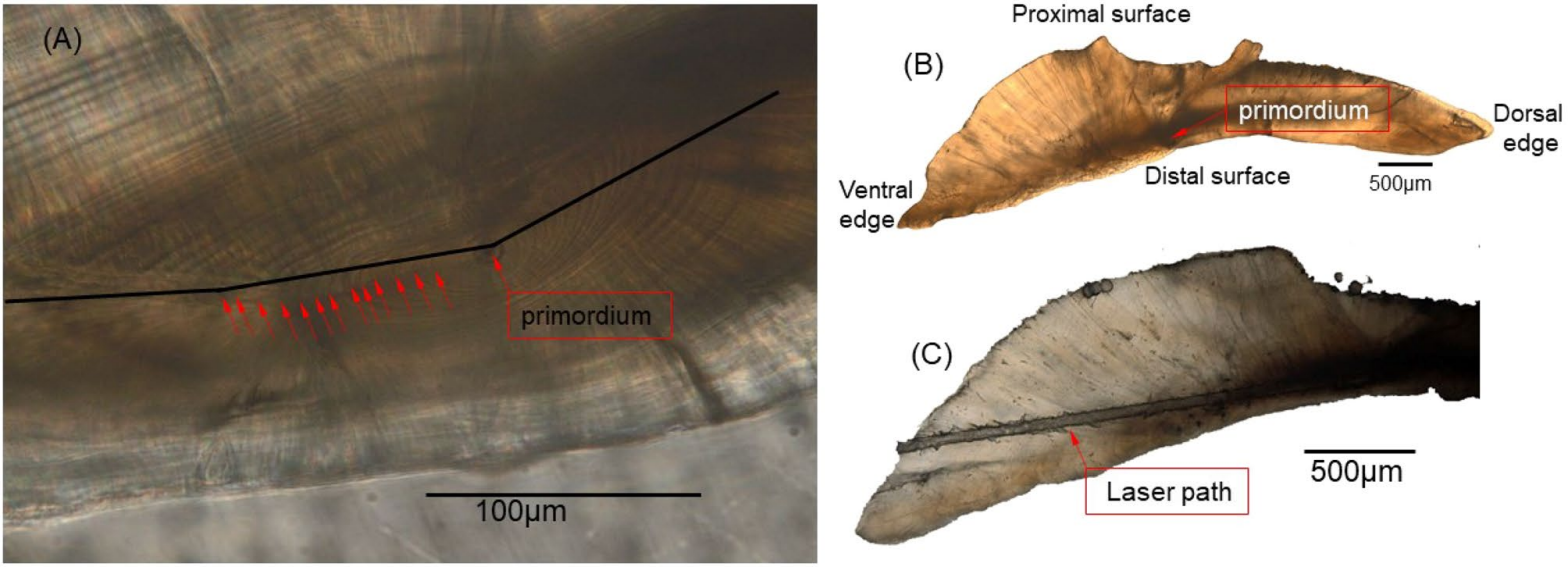
Polished otolith section of *Eleutheronema tetradactylum*. (**A**) Otolith section photograph reveal the primordium, red arrows indicate the daily increments, the black line indicates the center of the laser ablation path; (**B**) Complete graph of transverse section of a *E. tetradactylum* otolith; (**C**) The laser path on the otolith section.

The polished otoliths were visualized using an Olympus BX51 microscope (Olympus Co., Tokyo, Japan), which was equipped with an Olympus XC10 digital camera. The otolith primordium and daily increments of early life history were determined in the otoliths. By removing the two otolith that could not distinguish the primordium, the 64 otoliths were ultrasonically cleaned with ultrapure water (Millipore, Molsheim, France) for 5 min (Fig. 3).

Otolith element concentrations were obtained using a NW213 laser ablation system (New Wave Research, Fremont, USA) coupled with an Agilent 7500c inductively coupled plasma mass spectrometry (ICP-MS) (Agilent Technologies, Santa Clara, USA). Life-time elemental profiles were obtained from the core to the ventral-proximal otolith edge of each otolith by the line scanning ablation (wave length = 213 nm, pulse rate = 10 Hz, speed of sample stage moving = 10 μm/s, ablation diameter = 50 μm). Laser ablations occurred in a sealed chamber with an atmosphere of pure helium (He gas flow 800 mL/min). Blank background signals were measured for approximately 30 s before the analysis of each otolith to monitor and correct for instrument drift. At the beginning and end of the sampling for every 10 profiles, two certified standards MACS-3 (United States Geological Survey, USA) and NIST 612 (National Institute of Standards and Technology, USA) were ablated as external standards. Details of converted raw instrument intensity data to elemental concentrations are given in Mischel et al.^42^. The ^43^Ca was used as the internal standard, and concentration of ^43^Ca in otoliths was taken as 38.8% by weight or 388,000 ppm^43^. The elemental concentrations determined on the otoliths were converted to molar concentrations and standardized to calcium.

### Reconstruction of life history

This study was based on a well-supported assumption that throughout the lifespan of the fish individuals, the marked variations in Sr:Ca and Ba:Ca ratios could be associated with changes in water environment^14,20^. Therefore, habitat changes during the fish lifespan could be inferred from the fluctuation of otolith elementary profiles. The elementary time series from a fish moving between chemically distinct bodies of water could ideally be reduced to a piecewise stationary signal for capturing the marked change of environment^44^. The homogeneous Sr:Ca and Ba:Ca ratios within a segment were assumed to be associated with sedentary phases in similar habitats, the different segments split by MRT were assumed to represented the fish moving between heterogeneous environments^44,45^. The change points were screened by the chronological clustering based on multivariate regression tree (MRT) models carried out using the R package ‘Tampo’^44,46^.

Firstly, bivariate time series of standardized Sr:Ca and Ba:Ca ratios were screened to identify the marked element transect changes, and then partitioned to produce segments. Secondly, for each otolith bivariate time series, the means of Sr:Ca and Ba:Ca of each segment were calculated and used as the units for a k-means clustering analysis to determine the clusters of segments with similar Sr:Ca and Ba:Ca signatures. The k-means clustering was applied with the ‘NbClust’ package in R. The optimal number of clusters was assessed by Hubert index and D index through the ‘NbClust’ function of the ‘NbClust’ package. The ecological interpretation of clusters was supported by the previous study^28^. The segments in one cluster represented similar Sr:Ca and Ba:Ca signatures in a similar habitat environment. By contrast, the segments that were divided to different clusters corresponded to a distinct habitat. Finally, a transition matrix was used to represent the transitions of fish between different habitats. The time shares of individual spending in different habitats were represented by the length of the otolith ablation transect that the fish spent in each cluster, which may correspond to one or more segments.

### Life history pattern assignment

Distinct life history patterns were assigned by an unsupervised time series clustering algorithm based on the shape of their otolith element transects. The time-series clustering was performed based on dynamic time warping (DTW) distance and partitional clustering using the ‘dtwclust’ package. The time-series clustering based on DTW was used successfully in fish ecology to detect the subtle behavioral patterns from high resolution otolith microchemistry data sets^47^. Prior to DTW clustering, the Sr:Ca and Ba:Ca transects of each otolith were smoothed by a 5-points rolling average. To simplify the operations and speed up the algorithm, the transects were re-interpolated to a length of 300 cells, which was used as a rounded approximation of the mean length of series in the data set. The mean length of transects was 282 cells, standard deviation was 36, and maximum and minimum length were 399 and 203 cells, respectively. Due to the random start of partitional clustering procedures, it is a common practice to test the different random starts to evaluate several local optima and choose the best results out of all the repetitions. The generalized additive model (GAM) was used to fit the central tendency of the Sr:Ca and Ba:Ca profiles across different clusters, which was generated by the ‘mgcv’ package and plotted using the stat_smooth function of the graphics package ggplot2 within the R. Moreover, the age-at-transition from MRT were visualized by life sequences for each sample, and were grouped by life history cluster.

### Population structure

You et al.^48^ reported that the larval stage of *E. tetradactylum* lasted for 13 days after hatching, and the juvenile stage was kept from 14th day to 36th day. The laser pits were then each assigned to a life-history stage based on otolith increment analyses. Based on the images of the otolith increments in the early life, the laser ablation lines were assigned to core (corresponds to larval, the first 110 μm), near core (corresponds to juvenile, from 110-300 μm), and edge (adult, the last 20 μm, corresponds to 10 days before captured) areas. The average elemental concentrations of these areas were used to estimate the representative environments inhabited by each fish at the stages. We tested the differences of element concentration between different life stages using the non-parametric Kruskal–Wallis test. The pairwise differences of element:Ca values between populations and life stages were identified by the Dunn’s multiple comparison test.

Some elements were not used in the life history reconstruction because they may be affected by physiological factors and differed greatly at different ontogenetic stages. However, at the same ontogeny stage, the degree of physiological effects was similar, and these elements could also be used in population structure analysis to enhance the discriminative ability of otolith microchemistry data sets. Therefore, Li:Ca, Na:Ca, Mg:Ca, Mn:Ca, and Cu:Ca were included in the population structure analysis. Compared with other tropical fishes, larvae of *E. tetradactylum* displayed weaker swimming performance in terms of speed and endurance, thus the average elemental concentrations of the otolith core regions (the first 110 μm) were regarded as the signals of their natal origin. The otolith edge regions (the last 20 μm) corresponded to the sampling sites. We tested the differences of element concentration of core and edge between different sampling groups using the non-parametric Kruskal–Wallis test, the pairwise differences of element:Ca values between groups was identified by Dunn’s multiple comparison test.

Non-metric multi-dimensional scaling (nMDS) was used to illustrate graphically whether multi-elemental fingerprints had significant variations at different life stages among the sampling locations. The nMDS was performed with the ‘vegan’ package in R, the dissimilarity matrix was constructed based on Mahalanobis distances, and the stress value was used to assess the goodness-of-fit^36^. Since the movement of adult fish may cover a potentially large spatial scale, the collected regions did not necessarily correspond to the natal origin of the fish. Therefore, clustering analysis was conducted on the larval (natal) region of the otolith to explore the similarity between the specimens, to gain insights into the number of sources of adults and the connectivity between the sampling groups. The clustering analysis used an unsupervised learning algorithm that grouped together the samples with similar characteristics without prior classification. The best number of clusters was assessed by Hubert index and D index through the ‘NbClust’ function of the ‘NbClust’ package in R.

### Ethics declarations

All experimental protocols were approved by the Research Committee of City University of Hong Kong, Hong Kong. All methods were conducted in accordance with the relevant regulations and guidelines of the City University of Hong Kong. All methods are reported in accordance with ARRIVE guidelines.

## Results

### Population genetic structure

**Figure 4:**
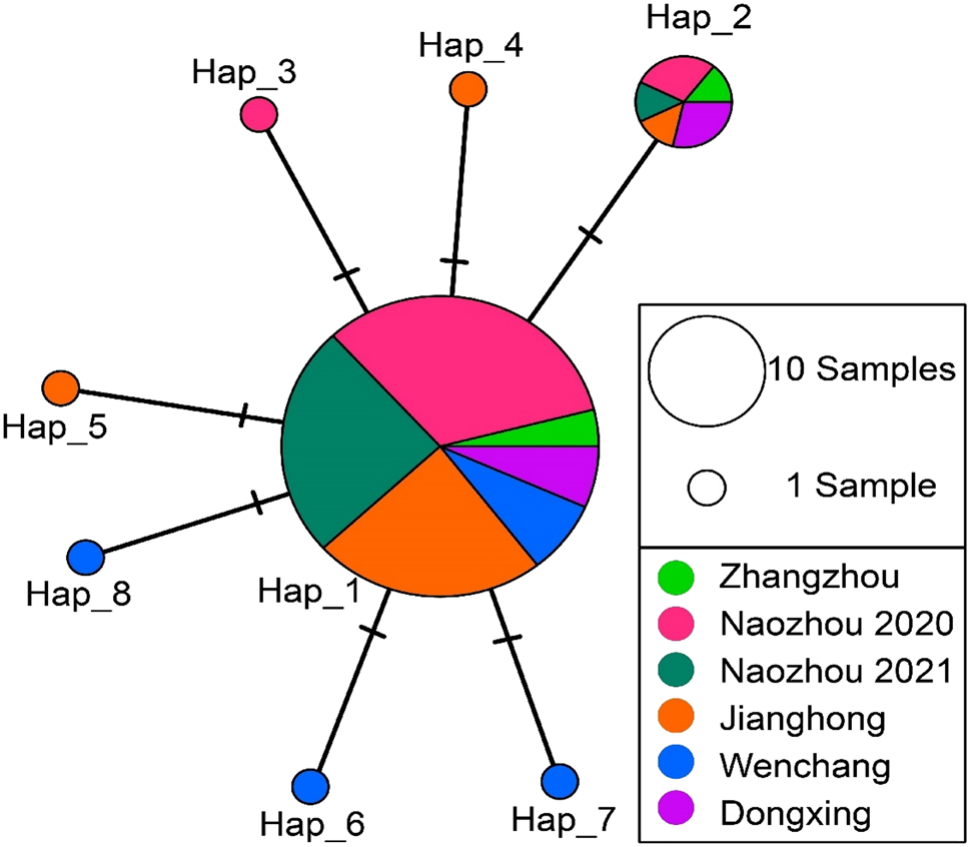
Median-joining network of haplotypes of the CO I gene sequences of *E. tetradactylum*. Each dash represents a single mutation step, haplotype frequency is related to the size of the circle. Different colors within the nodes refer to various sampling groups.

A total of 66 CO I sequences were successfully amplified and sequenced from *E. tetradactylum*, and the 66 sequences defined 8 haplotypes. A total of 7 polymorphic sites were identified in *E. tetradactylum*. Among these sites, there were 6 singleton variable sites, and only 1 parsimony informative site. The Hap 1 was the dominate haplotype, which included most *E. tetradactylum* individuals from this study, and was present in all sampling groups as shown in the haplotype networks (Fig. 4). The number and frequency of haplotypes in different sampling locations were in Table 2. The levels of population genetic differentiation between sampling groups were from −0.2404 to 0.1477, and all the pairwise FST between sampling groups were not statistically significant.

**Table 2:** Frequency of haplotypes in each sampling locations. Numbers indicate the number of individuals with this haplotype in the population.

	Zhangzhou	Naozhou 2020	Naozhou 2021	Jianghong	Wenchang	Dongxing
Hap_1	3	25	19	18	6	5
Hap_2	1	2	1	1	0	2
Hap_3	0	1	0	0	0	0
Hap_4	0	0	0	1	0	0
Hap_5	0	0	0	1	0	0
Hap_6	0	0	0	0	1	0
Hap_7	0	0	0	0	1	0
Hap_8	0	0	0	0	1	0

### Temporally resolved elemental ratios

**Figure 5:**
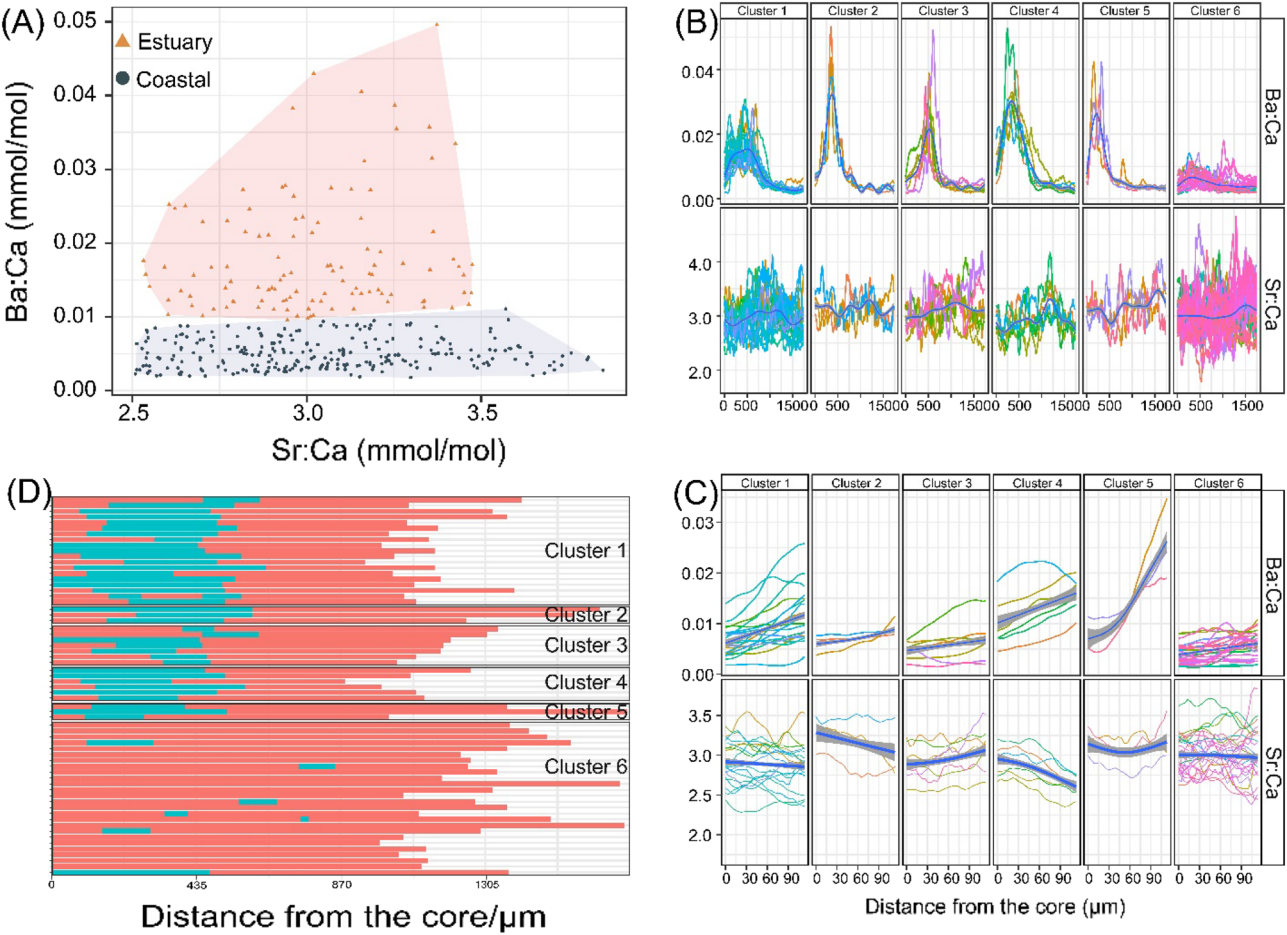
(**A**) Clustering of all of the otolith segments based on Sr:Ca and Ba:Ca elemental signatures of fourfinger threadfin collected in South China. (**B**) Partitional clustering based on Ba:Ca and Sr:Ca transects with dynamic time warping distance. Blue lines represent the fitted values by GAM. (**C**) The Ba:Ca and Sr:Ca data along otolith growth axes for larvae stage. (**D**) Individual life sequences of fourfinger threadfin inferred from the otolith elemental signatures (n = 64). Red bar: fish living in coastal habitat; blue bars: fish living in estuarine habitat. Samples were grouped according to the clusters shown in (B).

The MRT models based on the scaled Ba:Ca and Sr:Ca ratios detected the different numbers of splits. The number of segments identified from *E. tetradactylum* otolith Ba:Ca and Sr:Ca profiles ranged from 2 to 12, depending on individuals (on average 5.92 ± 2.29 segments). The hierarchical clustering based on the averaged Sr:Ca and Ba:Ca of each segments identified 2 chemically distinct clusters (Fig. 5A). Based on the previous study, these clusters correspond to two types of habitat environment. Segment cluster 1 featured intermediate Ba:Ca ratios (0.0098 < Ba:Ca < 0.0496 mmol/mol) and intermediate Sr:Ca ratios (2.53 < Sr:Ca < 3.47 mmol/mol), hereafter assigned to the ‘estuarine habitat’. The other cluster featured low Ba:Ca ratios (0.0018 < Ba:Ca < 0.0110 mmol/mol) with their widely distributed Sr:Ca ratios (2.51 < Sr:Ca < 3.83 mmol/mol), hereafter assigned to the ‘coastal habitat’. Along each transect on the otoliths, the assigned segments clustering to different types of habitats were used to reconstruct the life sequences and to estimate the amount of time spent in each habitat.

### Life histories patterns classification

From the unsupervised time series clustering approach, the element profiles of 64 fish were created into 6 clusters (Fig. 5B), based on the minimization of intra-cluster variance. For most *E. tetradactylum* individuals, significant fluctuations of the Ba:Ca profile occurred. However, the Sr:Ca ratios exhibited lower variability than Ba:Ca, even though there were some fluctuations in each profile but lack of general tendency. Cluster 1 (Fig. 5B, n = 19) included fish featuring intermediate Ba:Ca ratios during part of their early life history, with some segments assigned into estuary habitat. This Cluster was assigned to the ‘estuarine-dependent’ life history pattern. Moreover, the fish of Cluster 1 presented low Ba:Ca ratios at the larval and adult stages, corresponding to the segments that were assigned into coastal habitat. Cluster 2 (Fig. 5B, n = 3), Cluster 3 (Fig. 5B, n = 7), Cluster 4 (Fig. 5B, n = 6) and Cluster 5 (Fig. 5B, n = 19) contained individuals with a high Ba:Ca signal peak in their early life history, and the Ba:Ca dropped to a lower level although they started to decline at different times. The high Ba:Ca peak corresponded to the segments assigned into estuary habitat, indicating that these fish were reared in brackish or even in oligohaline waters (e.g., ‘estuarine-dependent’). Cluster 6 (Fig. 5B, n = 26) included fish featured with consistently low Ba:Ca ratios, and their profile segments were mainly assigned into the coastal habitat (i.e., ‘coastal-residents’ life history pattern). To show the details of the early life history stage (hatching to about 15–20 days), the first 120 μm of the element profiles was further separated (Fig. 5C). The fish of Cluster 1, 4 and 5 displayed a generally fast growth Ba:Ca ratios in larvae stage, in contrast to the relatively stable Ba:Ca ratios in Cluster 2, 3 and 6.

The time-at-transition from MRT were visualized by life sequences for each sample, and were grouped by life history clusters (Fig. 5D). Proportion of *E. tetradactylum* moving between different types of habitat environment were over 63.6%. Among all the fish samples, 12.1% of their first segment of profile was assigned into lower estuary habitat, and 19.7% of the samples had some segments that were assigned into upper estuary habitat. The experience of estuarine environments dominantly occurred in early life history stages, except the two samples of Cluster 6 (ZJ01 and ZJ31), which were considered to enter into estuary habitat at the beginning of the second half of life history.

### Population structure

**Figure 6:**
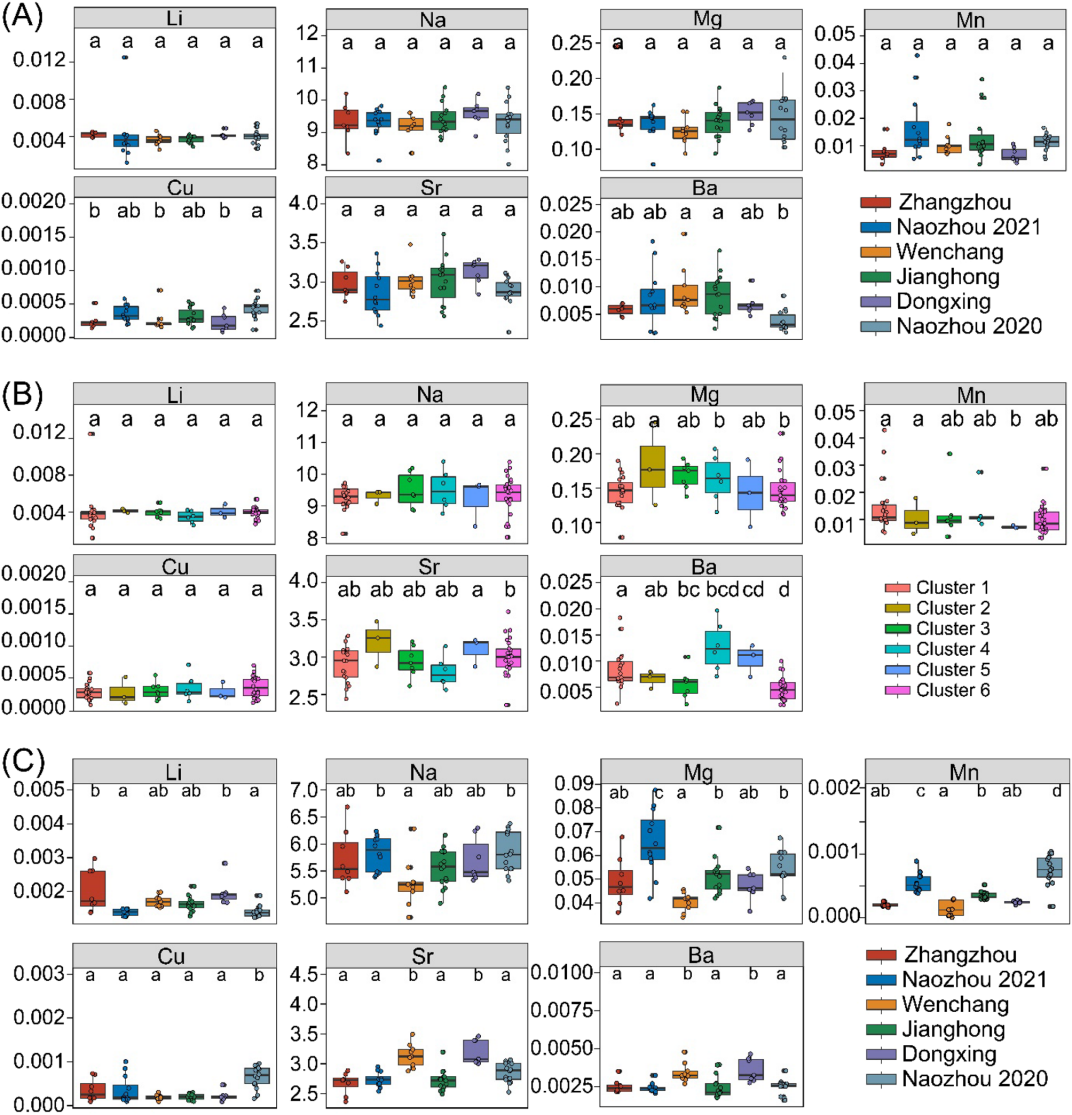
The mean element:Ca ratios of larval stage (**A**) comparisons of core data between different population, (**B**) comparisons between different clusters, and (**C**) comparisons of edge data between different populations. Different letters above the boxes indicate significant differences.

For the larval stage, the overall differences in mean elemental ratios among sampling locations were not significant (PERMANOVA,* p* > 0.05). Pairwise comparisons also showed no multivariate significant difference between any sampling locations pair. For single element, Li:Ca, Na:Ca, Mg:Ca, Mn:Ca and Sr:Ca ratios did not show significant differences (Kruskal–Wallis test; *p* > 0.05). Cu:Ca and Ba:Ca only showed highly significant differences among few locations (Kruskal–Wallis test, *p* < 0.05) (Fig. 6A).

Between the different clusters of life sequences, at the larval stage, the overall differences in mean elemental ratios among sampling locations were also not significant (PERMANOVA, *p* > 0.05). Pairwise comparisons showed no multivariate significant difference between any clusters. For single element, Li:Ca, Na:Ca, Mg:Ca, Mn:Ca, and Cu:Ca ratios were not significantly different (Kruskal–Wallis test; *p* > 0.05). Only Ba:Ca and Sr:Ca showed highly significant difference among a few clusters (Kruskal–Wallis test, *p* < 0.05) (Fig. 6B).

For the otolith edge, the overall differences in mean elemental ratios among sampling locations were significant (PERMANOVA,* p* < 0.05). Pairwise comparisons showed multivariate significant difference between any sampling locations pair. For single element, all elements showed significant differences among locations (Kruskal–Wallis test, *p* < 0.05) (Fig. 6C).

**Figure 7:**
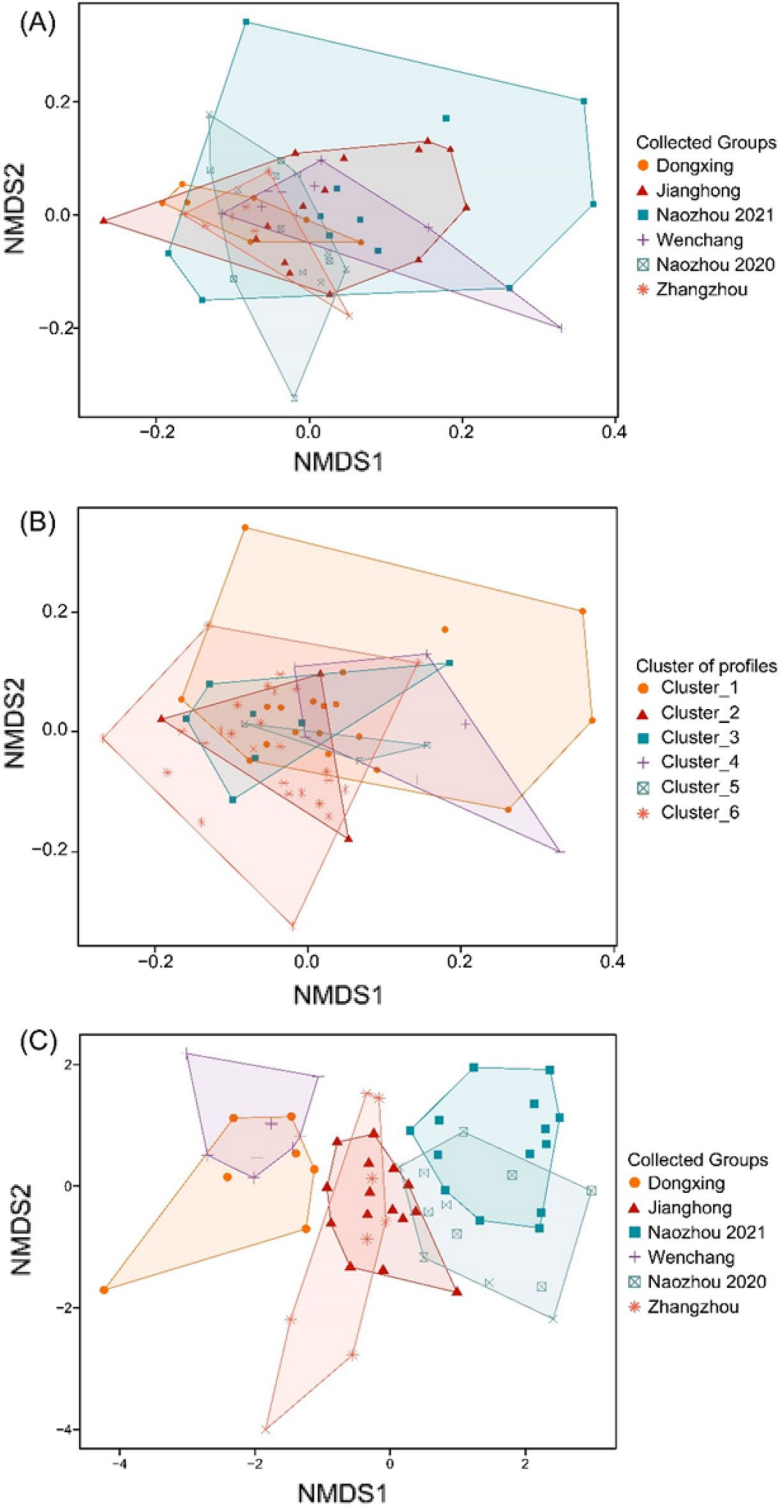
Relationships between fourfinger threadfin individual using nonmetric multidimensional scaling. For otolith core element signals, sampling locations and cluster of profiles were respectively identified by colored shapes in (**A**) and (**B**), and for otolith edge element signals, sampling locations were identified by colored shapes in (**C**).

Based on the plot of two-dimensional nMDS, it was obvious that individuals from different sampling locations were difficult to be discriminated (Fig. 7A). The two-dimensional nMDS showed that individuals assigned to different clusters were also difficult to be discriminated by their core chemical signals (Fig. 7B). However, for the otolith edge data, nMDS showed that individuals from different sampling locations were divided into three separate clusters. The samples of Naozhou from different years were closed (Fig. 7C).

**Figure 8:**
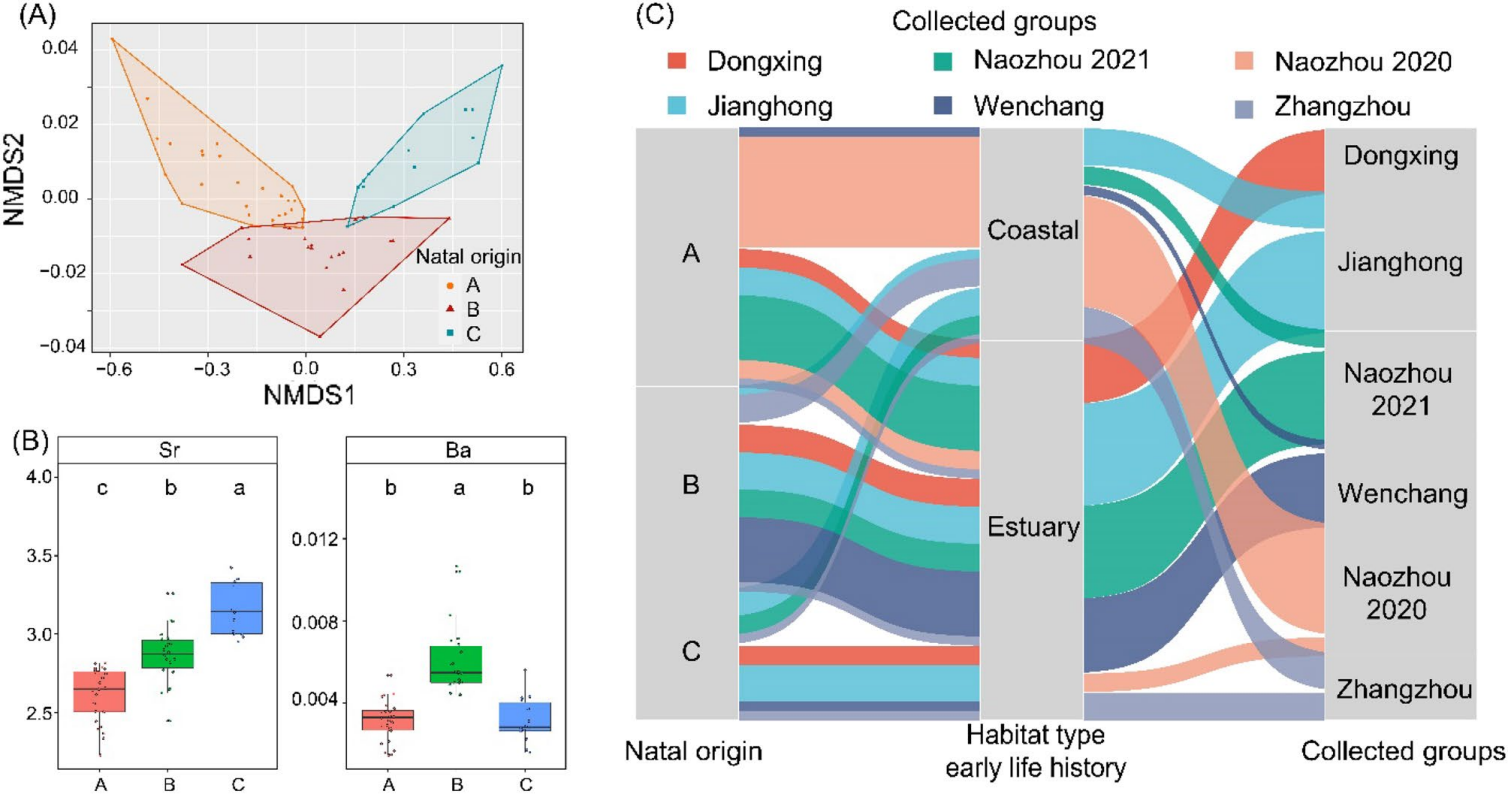
(**A**) Clustering based on otolith chemistry in the larval period for fourfinger threadfin. (**B**) Comparison of dominant clusters (**A**–**C**) based on otolith chemistry in the larval period. (**C**) Frequency distribution of fourfinger threadfin (n = 64) by cluster for the three natal origin, two habitat type and six collected groups studied. The height of each box represents the proportion, the size of color lines indicates the proportion of individual.

Because the distributions of Li:Ca, Na:Ca, Mg:Ca, Mn:Ca, and Cu:Ca ratios in each collected group and Cluster of profiles were not significantly different, these elements were not used in clustering analysis. Clustering analysis of the larval (natal) region of otolith identified 3 clusters (names A, B and C) of chemically distinct larval elemental signatures (Fig. 8A). The overall differences in mean elemental ratios between the two natal origin clusters were significant (PERMANOVA, *p* < 0.05). Sr:Ca and Ba:Ca were significantly different (Kruskal–Wallis test; *p* < 0.05). The natal origin A with low Sr:Ca and low Ba:Ca, may correspond to spawning grounds out of the estuarine habitat. The natal origin B was characterized by higher Ba:Ca ratios than the other two natal origin clusters, corresponding to spawning ground close to the estuary. The natal origin C was characterized by higher Sr:Ca ratios than the other two clusters, corresponding to the spawning ground far away from the estuary (Fig. 8B).

For each sample, the natal origin and life history pattern that were assigned to and the location of fish collection were all visualized by the alluvial plot, to gain insights into the sources of adults and connectivity between sampling groups in the present study (Fig. 8C). The natal origin A contained 28 samples and contributed to more than half of the coastal-dependent type. Thirteen of 28 grew up in the coastal habitat, and 15 of 28 utilized the estuary habitat in their early life history. All the fourfinger threadfin from Naozhou 2020 were clustering into natal origin A, even though they had different life histories. The natal origin B included 22 samples, among which four grew up in the coastal habitat, and 18 utilized the estuary habitat in their early life history. The natal origin C included 14 samples, among which 6 grew up in the coastal habitat and 8 utilized the estuary habitat in their early life history. The samples collected from GX all utilized the estuary habitat in their early life history, whereas other groups contained both estuary and coastal habitats in their early life history. The dominant early life history types of Naozhou 2020 group and Naozhou 2021 were different.

## Discussion

**Figure 9:**
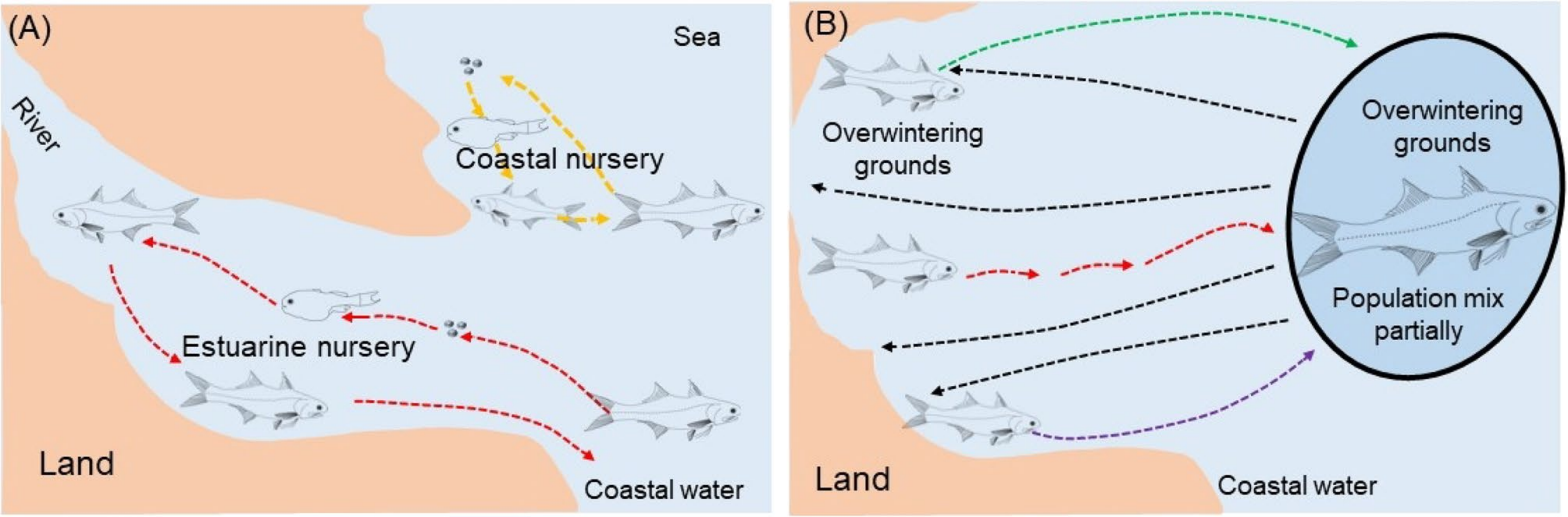
Diversity of early life history and population connectivity. (**A**) Diagrammatic model of life history of estuarine nursery type and coastal nursery type. (**B**) The juvenile subpopulations from different natal nurseries partially mix at the overwintering grounds, and then migrate back to the coastal habitats. The green, red, purple arrows indicate the fish migrating from natal nurseries to overwintering grounds, and the black arrow indicates the part of mixed fish population back to different coastal habitats.

Effective management strategies for *E. tetradactylum* in Chinese coastal waters require an understanding of population structure, connectivity, and migration patterns of the disparate spawning components. This study used otolith microchemistry to evaluate the elemental signatures of entire life history of the fish individual. A multivariate partitioning, MRT, splits the nucleus-edge microchemistry transects into partitions by detecting the discontinuity, thus dividing the transects into distinct segments with significant differences. The results showed that these fragments could be assigned to two clusters, and corresponded to different habitat types. The otolith core elements were clustered into three clusters (A, B, C), possibly corresponding to three spawning grounds with different water environmental characteristics. However, the possibility of more spawning grounds cannot be completely ruled out, although some of them may have similar chemical signatures. Finally, among all the sites in this study except NZ 2020, samples were derived from three different spawning grounds, indicating the admixture of the entire population. Figure 9 illustrates the diversity of early life history and population connectivity of threadfin fish.

### Reconstruction and diversity of life history

According to the clustering analysis for the entire otolith element profiles, the 64 individuals were divided into 6 clusters. However, based on the pattern of life sequences they could be mainly divided into two modes, namely estuarine nursery type and coastal nursery type. For most individuals, the Ba:Ca ratios near the core of the otolith (corresponding to the embryonic stage to 10–14 days after hatching) were lower than 0.008, with an overall content < 0.011 and Sr:Ca ratio between 2.4–3.5, indicating that the hatching of fertilized eggs and planktonic larvae were mainly in the nearshore seawater. Previous studies showed that the optimum salinity for *E. tetradactylum* hatching was 25–35^48^, consistent with the results of this study.

After the formation of the caudal fin, *E. tetradactylum* gradually increased its swimming ability. The larvae were able to swim across 40 km at 10 cm/s when they reached a standard length of 17 mm^49^, meaning that larvae (10–15 days, about 20 mm standard length) were able to reach the settlement habitat. In this study, the otolith Ba:Ca ratios increased significantly at the beginning of juvenile stages for those individuals accessing to the estuarine nurseries (Fig. 5C). Whereas, the otolith Ba:Ca ratios did not fluctuate significantly for individuals that settled in shallow coastal waters. Our results showed that 57.8% of the individuals entered the estuarine nursery from the pelagic environments, with the initial ingresssion from ocean to estuary occurred within approximately 15–20 days after hatching. This was consistent with the transition period from larvae to juvenile^48^. After juveniles spent varying amounts of time in the estuarine nursery, they gradually moved into nearshore marine habitats.

Ba:Ca ratios across a freshwater-seawater salinity gradient typically displayed an exponential decline, with the lowest ratios in seawater end-members^50,51^. Therefore, the Ba:Ca ratio of adult *E. tetradactylum* entering the marine environment was very low when compared with the juvenile stage in estuarine nursery, and only a small number of individuals showed low Ba:Ca peak in adult stage (Fig. 5B). The low Ba:Ca peak in adult stage may be the imprint of those adults entering the estuarine environment. Some previous studies suggested that some threadfins moved into estuarine habitats^9,52^. We also found the remnants of freshwater fish and threadfin juveniles in the stomach of adult *E. tetradactylum*s. These threadfin fish may experience marine and estuarine or even freshwater environments. Therefore, the transition between marine and estuarine and even freshwater environments likely persisted throughout the life history of *E. tetradactylum*, as recorded by the increased otolith Ba:Ca ratio. Moreover, the height of Ba:Ca peak in adult stage was lower than that in juvenile stage, which may reflect the decrease in the rate of accretion otolith material and the resultant compression of growth increments as fish age. The laser beam ablated the otolith material at a fixed speed, and each second of ablation integrated an increasingly greater period of accretion in the later increments of older fish, thus the resolution of temporal chemical variation for older fish decreased^7,53^. Thus, the elevated Ba:Ca ratio brought by the short-term estuarine life was averaged by the long-term seawater low Ba:Ca ratio. Additionally, changes in the environment may be relatively rapid as evidenced by the otolith composition (detected within 2–3 d by Miller^54^, and 10 d by Yokouchi et al.^55^). The otolith composition did not stabilize for 12–14 d, indicating that habitat transitions may be discernable within a short period of time, with a complete reflection of new water by otolith for up to 2 weeks to 1–2 month^54,55^. For example, older adult fish may still migrate into estuarine habitats, but only for a shorter period of time than that required for estuarine elemental signatures.

On the other hand, the low Ba:Ca peak in adult *E. tetradactylum* otoliths may be attributed to the increased Ba in the ambient seawater, possibly sourced from terrigenous materials and upwelling. Locations with strong upwelling activity have elevated Ba:Ca ratios in surface waters^56^. The regional upwelling off the Hainan and Guangdong coast effectively enriched the nutrient concentrations in the water^57^, and thus may cause high Ba:Ca ratio in adult *E. tetradactylum* otoliths. Moreover, the Kuroshio intrusion could bring dissolved barium with high concentration. In the surface layer of the East China Sea, the Ba concentration was the highest in coastal water and gradually decreased seaward^58^. However, the Ba concentration of otolith edge of adult fish from Zhangzhou was not higher than that in other sites. Therefore, although the elements at the edge of the otolith showed differentiation, the lack of information about the elemental composition of ambient seawater made it difficult to link the difference in otolith elements with environmental variables. If the water samples can be collected at the same sampling sites of fish for physical and chemical analysis, it may be able to conduct correlation analysis between otolith elements and water samples^30^.

The diversity of life history was also reflected by the fact that most adult fish caught in Zhanjiang in 2020 utilized the coastal nursery and few of them entered the estuarine nursery. However, it is interesting that the fish captured from the same sampling sites in 2021 mostly utilized estuarine nursery habitats. Previously, we demonstrated two contrasting environmental history patterns of *E. rhadinum* and *E. tetradactylum*^28^. Specifically, most analyzed *E. rhadinum* individuals entered the estuarine nursery, whereas the majority of *E. tetradactylum* individuals utilized coastal nursery. In this study, we found that the dominant life-history strategy in *E. tetradactylum* individuals changed. The relatively few individuals from the coastal nursery in this study were rather unexpected from this study.

This phenomenon may reflect the interannual fluctuations in the advantages of estuarine nurseries. Estuaries are important nursery areas for numerous fishes with highly dynamic habitats, and susceptible to significant alteration by both natural and anthropogenic activity^37,59,60^. Estuarine nurseries have many advantages, such as the turbid water avoiding predation, salinity gradient habitats, higher primary productivity and subsequent food availability^36^. The fluctuation of freshwater input may lead to changes in estuarine salinity and productivity. In some cases the high flows may have a negative impact (e.g. *Micropogonias furnieri* in the Río de la Plata estuary, Argentina–Uruguay^61^), whereas in other cases the high flows may have a positive impact (e.g. *Polydactylus macrochir* in a dry-tropical estuary^39^ and *Argyrosomus japonicus*, in eastern Australia^36^). Based on the total length of samples, we estimated that the ages of *E. tetradactylum* samples were at the same age (4 +). Therefore, the samples of Naozhou 2020 were hatched in 2016, and the samples of Naozhou 2021 were hatched in 2017. The breeding time of *E. tetradactylum* in Southern China is mainly from April to May, thus their larval and juvenile stages were mainly from May to July. The monthly average runoff data (Fig. 2) indicated that in coastal basin of southern China, runoff of June and July in 2016 was lower than that in 2017, although this was not statistically significant. When the freshwater runoff decreases, the slimming down of estuarine nurseries areas or the decline of the advantages of estuarine nurseries may lead to the lower proportion of estuarine-utilized *E. tetradactylum* in the whole population. This may explain the low proportion of estuarine nursery type in Naozhou 2020 samples. However, this interpretation was only based on the sparse data available for two years and one location. Further long-term fishery resource monitoring and early life history investigation are still needed.

It can be confirmed that *E. tetradactylum* has a diversity of life history patterns and more importantly, the importance of life history diversity for species survival and resilience^8^. Facing the declining habitat availability due to climate change or anthropogenic impacts, the single life-history pattern was prone to interruption of their complex life history with a lack of available habitat, leading to local population collapse or extinction. In contrast, if the species accommodated multiple life-history patterns, other habitats can ensure population continuation even though the availability of one habitat is limited.

Similar intraspecific variability in juvenile habitat use and ontogenetic habitat shifts^62,63^ was also observed in other studies based on otolith microchemistry. Diversity in life history patterns may indicate a bet hedging strategy with the goal of reducing the risk through the averaging of independent random events^8,64^. Different phenotypes increase the probability with at least some offspring thriving under different possible conditions, leading to a higher fitness and reducing the likelihood of extinction^65^. Intraspecific variability in habitat use can increase the likelihood for larvae and juveniles to distribute in suitable habitats. Such strategy is critical for the resilience of fish stocks living in dynamic near-shore and estuary environments^64^.

### Population connectivity

Population productivity tends to be highly dependent on the connectivity between the adult population and juvenile subpopulations which utilize different nurseries^4,66^. In addition, connectivity and well-timed migration between habitats during different ontogenetic stages are often beneficial to fish^4,62^. In this study, we found the habitat connectivity between spawning ground and different nurseries, and the interannual variation of connectivity between pelagic environments and the estuarine habitats. The proportion of estuarine nursery utilized in adult fish captured in 2021 was higher than that in 2020. This might be the result of the changes in environmental factors (e.g., temperature, salinity, dissolved oxygen, and turbidity) that could affect species productivity, recruitment success, relative abundance, and distribution^36,67,68^. Estuaries are transitional zones linking freshwater and fully marine environments, and sensitive to the effects of climatic conditions and the degree of freshwater inflow. Various mechanisms such as water physico-chemistry, nutrient levels, transport or retention of larvae, and changes in availability of suitable habitats may affect the survival of larvae in estuarine nurseries^36,65,66^. These mechanisms are not be mutually exclusive and operate over differing temporal and spatial scales. In general, the highly-variable year class likely resulted from variable environmental conditions in different years, and this phenomenon were also observed for some estuarine-dependent fishes^62,69^.

Along the southern coast of China, the impact of urbanization and human activities caused considerable changes in the coastal landscape. Protecting habitats such as estuaries and coastal intertidal zones and their connectivity is important for the survival of *E. tetradactylum* during their early life history stages. For adult fish collected from different locations, both mitochondrial genetics and otolith core element analysis showed that *E. tetradactylum* from Southern China coastal environments displayed mixed population structures at the adult stage. Cluster analysis of otolith core elements showed that there were three spawning grounds with different physicochemical environments, and larvae entered different nurseries at the end of pelagic development, and then recruited to different adult populations. The collected adult fish populations were distributed over a range of hundreds of kilometers or even thousands of kilometers. The mixed population structure at this level of distance cannot be fully explained by the dispersal capacity of larvae, and was dependent on ocean currents to dispersal to new locations. The directionality of ocean currents (GDWCC and GDCC) was not feasible to mix the larvae from different spawning grounds along distant routes of transmission^34,35^. Therefore, the dispersal more likely occurred after the young fish were recruited to the adult populations. In the wintering stage, *E. tetradactylum* populations of China from different spawning grounds and nurseries may exist as a form of mixed stock in a warm ocean, afterwards fish schools from different spawning grounds may not migrate back to their natal habitat.

*E. tetradactylum* collected from Australia showed strong isolation by distance, indicating limited mixing and therefore long-term separation of post-juvenile fish, even within a range of 15 kilometers^70–73^. This may be due to the different climatic types in the distribution areas of *E. tetradactylum* in Southern China and Australia (i.e. subtropical and tropical). *E. tetradactylum* were not able to tolerate lower temperatures, and begins to die when the water temperature drops below 14 ℃. Along the southern coast of China, the monthly average water temperature in winter may be lower than 17 ℃, while the monthly average water temperature of the sea near Hainan is above 23℃ for the full year. The water temperature in the distribution area of *E. tetradactylum* in Australia is above 22 ℃ for the full year. Therefore, *E. tetradactylum* in China may conduct overwinter migration and overwinter in the waters near Hainan and even the coast of Vietnam where the temperature is suitable. However, *E. tetradactylum* in Australia may not have overwintering behavior. Of course, whether the *E. tetradactylum* in China have overwintering migration requires further study, such as using pop-up satellite archival tags (PSATs)^74^to study the habitat utilization and large-scale migration of adult *E. tetradactylum*.

Otolith microchemical signatures showed that adult individuals from the same sampling locations may originate from different spawning grounds, demonstrating mixed population structure along the coast of China. Moreover, the otolith core chemical signature was able to theoretically identify the contribution of an external source to the fishing grounds, and the three potential sources found in this study showed different levels of contribution to the adult population. However, intrinsic (i.e., ontogenetic effects) processes may affect the chemical incorporation into otoliths^75,76^. Although there are obvious differences in the elemental characteristics of the otolith edge in different sampling sites, the possible influence of ontogenetic stages on otolith elements constrained the comparison of otolith core data with edge data to infer the location of possible spawning grounds. Therefore, the three possible spawning grounds inferred by the core of adult fish were difficult to be correlated with the sampling sites. The otolith core element fingerprints of adult individuals cannot provide direct information on the accurate location of the different spawning grounds and/or larval sources. To estimate the accurate natal origin of the adult fish, it is necessary to establish a multi-element birth baseline dataset for larvae and/or juveniles, which needs sampling pre-dispersal life stages of known origin, and all spawning grounds or nursery areas need to be sampled.

To conclude, otolith microchemistry appeared to be an effective method to investigate the life history and population connectivity of *E. tetradactylum* in the South Coast of China. Our results demonstrated the diversity of life history patterns of *E. tetradactylum*, with both estuaries and coasts serving as key nursery habitats after the larvae left pelagic environments. After migrating away their nursery habitats, the juveniles appeared to mix when they fed and overwintered in extensive seawaters, and did not exactly return to their natal habitat after overwintering, resulting in a diversity of possible sources within each sampling location, and lack of structure. The high levels of life-history connectivity implied that the restoration in egg and larvae densities of *E. tetradactylum* in coastal waters and estuaries may enhance their population abundance.

## Data Availability

The datasets generated and/or analyzed during the current study are available in the NCBI Genebank repository, Access no. OP882686 - OP882693.
